# Spatio-temporal modelling and prediction of malaria incidence in Mozambique using climatic indicators from 2001 to 2018

**DOI:** 10.1038/s41598-025-97072-6

**Published:** 2025-04-08

**Authors:** Chaibo Jose Armando, Joacim Rocklöv, Mohsin Sidat, Yesim Tozan, Alberto Francisco Mavume, Maquins Odhiambo Sewe

**Affiliations:** 1 Department of Public Health and Clinical Medicine, Sustainable Health Section Umeå University, Umeå, Sweden; 2 Heidelberg Institute of Global Health and Interdisciplinary Centre for Scientific Computing, Heidelberg University, Heidelberg, Germany; 3 Faculty of Medicine, Eduardo Mondlane University, Maputo, Mozambique; 4 School of Global Public Health, New York University, New York, USA; 5 Faculty of Science, Eduardo Mondlane University, Maputo, Mozambique; 6 Heidelberg Institute of Global Health, Heidelberg University, Heidelberg, Germany; 7 Center for African Studies, Eduardo Mondlane University, Maputo, Mozambique

**Keywords:** Malaria, Mozambique, Early warning, Climate, Prediction, Climate sciences, Environmental sciences

## Abstract

Accurate malaria predictions are essential for implementing timely interventions, particularly in Mozambique, where climate factors strongly influence transmission. This study aims to develop and evaluate a spatial–temporal prediction model for malaria incidence in Mozambique for potential use in a malaria early warning system (MEWS). We used monthly data on malaria cases from 2001 to 2018 in Mozambique, the model incorporated lagged climate variables selected through Deviance Information Criterion (DIC), including mean temperature and precipitation (1–2 months), relative humidity (5–6 months), and Normalized Different Vegetation Index (NDVI) (3–4 months). Predictive distributions from monthly cross-validations were employed to calculate threshold exceedance probabilities, with district-specific thresholds set at the 75th percentile of historical monthly malaria incidence. The model’s ability to predict high and low malaria seasons was evaluated using receiver operating characteristic (ROC) analysis. Results indicated that malaria incidence in Mozambique peaks from November to April, offering a predictive lead time of up to 4 months. The model demonstrated high predictive power with an area under the curve (AUC) of 0.897 (0.893–0.901), sensitivity of 0.835 (0.827–0.843), and specificity of 0.793 (0.787–0.798), underscoring its suitability for integration into a MEWS. Thus, incorporating climate information within a multisectoral approach is essential for enhancing malaria prevention interventions effectiveness.

**Supplementary Information:**

The online version contains supplementary material available at 10.1038/s41598-025-97072-6.

## Introduction

Accurate and timely predictions of malaria incidence is important for the implementation of effective interventions to reduce morbidity and mortality in vulnerable populations. Malaria is among the most prevalent and serious tropical diseases that require precision in response tailored to the local situation^1,2^. Malaria remains a significant global health challenge, with an estimated 229 million cases and 409,000 deaths reported in 2019^3^. In 2020, the burden of the disease increased to 241 million cases and approximately 627,000 deaths^4^. By 2021, the number of cases increased further to 247 million with an estimated 619,000 deaths^5^. The upward trend continued in 2022, with malaria cases reaching 249 million and approximately 608,000 fatalities^6,7^. In Sub-Sahara Africa, nearly 233 million malaria cases and 580,000 fatalities were reported in 2022^3^. Malaria is endemic throughout Mozambique, with approximately 6 million cases reported each year^4^. The transmission intensity varies from year to year and region to region, modulated by climate and socioeconomic factors. In Mozambique, *Anopheles funestus sensu* stricto is the major mosquito vector at 95%, with *Plasmodium falciparum* being the predominant *Plasmodium* species, responsible for 90% of all malaria cases^5–7^. Malaria transmission is highly seasonal and varies considerably across different regions in Mozambique, with the highest cases being observed during the rainy season from November to April^8,9^.

Efforts to reduce the malaria burden and deaths among vulnerable populations should involve a clear control strategy, effective resource mobilization, active community engagement, and the integration of a Malaria Early Warning System (MEWS) in order to strengthen planning, resource optimization, and community empowering to achieve effective malaria control^10^. Mozambique has undertaken several malaria eradication initiatives over the years, including the Global Malaria Eradication Program (1960–1969), the Lebombo Spatial Development Initiative (1999–2011), and MOSASWA regional initiative and the Mozambican Alliance Towards the Elimination of Malaria (MALTEM) established in 2015. These initiatives primarily focused on indoor residual spraying (IRS) and the distribution of insecticide-treated nets (ITNs) by the country, later incorporating artemisinin-based combination therapies^11^. Recent efforts, such as the Magude initiative, adopted a comprehensive approach that integrated indoor residual spraying (IRS), insecticide-treated nets (ITNs), mass drug administration, and community engagement to strengthen malaria control, resulting in a reduction in malaria prevalence from 33% in 2005 to 23% in 2011^12,13^. Insights from these past efforts are critical for shaping future strategies in Mozambique.

Incorporating climate information into a multisectoral approach is crucial for enhancing malaria interventions by enabling more accurate predictions of outbreak risks and optimizing the timing and targeting of control measures^14^. Climate factors such as precipitation and temperature are crucial for malaria prediction due to their direct biological impact on the disease’s transmission dynamics^15^. Several studies have used climate variables to predict malaria incidence, including a study in Kenya that developed two distinct models using satellite data on land surface temperature (LST), evapotranspiration (ET), and precipitation to forecast monthly malaria incidence with a 1–3-month lead time, finding that the GAMBOOST model demonstrated the highest predictive accuracy at a 1-month lead time and is suitable for inclusion in a malaria early warning system^16^. A study in Ethiopia found that rainfall with a 1–2 month lag significantly affected malaria incidence around large dams, while minimum and maximum temperatures with the same lag were significant only at highland sites^17^. Ototo et al.^18^ found that in the Lake Victoria Basin, historical malaria cases are positively and linearly correlated with 3–6 month averages of monthly rainfall and maximum temperatures, based on regional climate projections. Adeola et al.^19^ used time-series data to analyze malaria incidence and environmental factors in South Africa, revealing that rainfall is the key determinant of malaria cases and showing that their SARIMA model could forecast malaria incidences up to 3 months in advance. Pillay et al.^20^ employed a novel high-resolution malaria dataset and a deep learning transformer model for climate-informed predictions, demonstrating that the model can effectively provide short-term forecasts for the Limpopo area from 2 to 16 weeks in advance. The findings highlight the importance of integrating climate information into malaria early warning systems, as climate and surveillance data are essential for developing effective alerts and enhancing public health interventions.

In countries like Mozambique, where many areas are hyper- and holo-endemic for malaria, defining “outbreaks” is challenging for several reasons, including unavailability of accurate and timely data to chart accurate endemic channels due to deficiencies in existing health information systems. Additionally, not all cases of malaria are captured within the national health services due to alternative health care providers including traditional healers and other informal providers. Thus, it is well recognized the necessity to establish a more robust health information and surveillance systems to detect eventual outbreaks of malaria in hyper- and holo-endemic areas.

In 2001, the WHO introduced a framework for establishing MEWS in Africa, based on three indicators: vulnerability, transmission risk, and early detection. Vulnerability indicators encompass factors such as socioeconomic status, education level, immunity, malnutrition, HIV status, proportion of pregnant women and children under 5 years of age. Transmission risk indicators include climatic factors such as anomalies in in precipitation or temperature. Early detection indicators are threshold based indicators derived from routine surveillance data that are crucial in detecting early onset of an outbreak^21^. While such frameworks provide valuable tools, their adaptation to regions with continuous high transmission requires careful consideration. For endemic areas like Mozambique, the concept of “outbreaks” may need to be reframed to account for the persistent baseline and focus on detecting unusual deviations from the seasonal norm. This approach ensures the framework remains relevant and actionable in endemic settings.

Thresholds for malaria epidemics can be calculated in various ways, one of which involves determining the long-term average of monthly malaria cases over at least 5 years of data, while excluding any outliers. The epidemic threshold is then set at two standard deviations above this mean, which helps to identify significant increases in malaria incidence. This approach, endorsed in Africa due to its success in Thailand in the early 1980s, helps in predicting malaria outbreaks^22^. Several studies have explored the use of MEWS by integrating climate factors, such as temperature, rainfall, and relative humidity, to improve predictions of malaria outbreaks^23^. A study conducted in Ethiopia developed the EPIDEMIA system to combine weekly malaria surveillance with environmental data, automating forecasting, reporting, and risk mapping to improve malaria outbreak prediction. EPIDEMIA marks a significant advance in malaria surveillance by delivering timely, actionable information for effective public health responses^24^. Githeko et al.^25^ found that rising anomalies in maximum temperatures and rainfall in Kenya are associated with increased malaria incidence, prompting the creation of a prediction model for malaria epidemics. Evans et al.^26^ developed a hyper-local malaria early warning system for rural Madagascar, combining malaria case data with high-resolution environmental data to forecast malaria incidence and medical supply needs up to 3 months ahead, outperforming previous models and showing promise for broader use with digitized health data.

A lag period in malaria early warning systems is the delay between climatic conditions and the rise in malaria incidence, which is crucial for predicting future outbreaks and ensuring that interventions, such as deploying medical resources or implementing control measures, are timely and effective, thereby enhancing the system’s ability to prevent or reduce malaria outbreaks. Identifying lag periods of climate factors, and adequate model are some of the key steps before an effective disease prediction can be developed based on local environmental information. Predictive models for malaria transmission have been investigated in several low-resource settings. In South Africa, the distributed lag nonlinear modeling (DLNM) framework was employed to predict malaria cases using precipitation and ambient temperature.

The study’s findings reveal that seasonal climate forecasts effectively matched short-term malaria predictions with observed cases, underscoring the importance of incorporating climate data into malaria forecasting to enhance prediction accuracy and facilitate timely responses^27^. A study in Uganda used a Random Forest model to predict malaria incidence rates with high accuracy, achieving an R^2^ of 0.88 and a Mean Squared Error (MSE) of 0.0534. Antimalarial treatment was the most influential factor in reducing cases, while mosquito net access significantly lowered rates. Higher temperatures were found to correlate with increased malaria incidence, highlighting the critical role of both climate factors and preventive measures^28^. MEWS are important for risk management and detect anomalies in malaria infection based on environmental conditions in low- and middle-income countries^29,30^. A Malaria Early Warning System (MEWS) can serve as a crucial tool for policymakers, providing actionable insights into ongoing and anticipated outbreaks in Mozambique. However, the system currently under development in the country is primarily designed for short-term forecasting, with prediction windows limited to 4–8 weeks^31^.

Unusual increase of climate factors such as precipitation, temperature, relative humidity, and NDVI can be used to confirm the onset of an epidemic. A deeper comprehension of the climatic factors influencing malaria transmission can help anticipate potential changes in disease patterns and formulate strategies for preventive action in Mozambique. This study aims to develop and evaluate a spatial–temporal prediction model for monthly malaria incidence in Mozambique for potential use in a malaria early warning system.

## Results

**Fig. 1:**
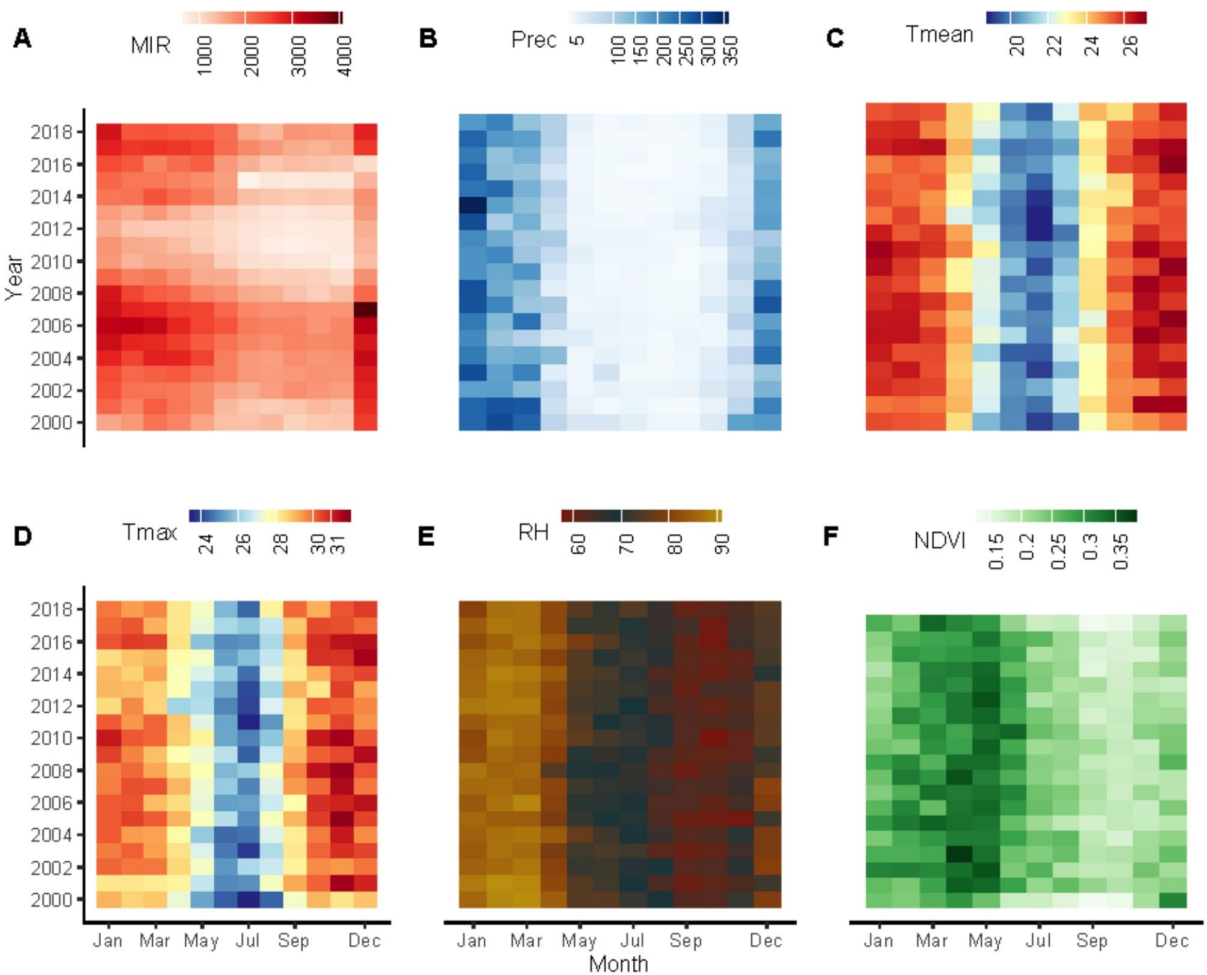
Annual cycle of (**A**) malaria incidence rates (per 100,000 population) in Mozambique, (**B**) Precipitation, (**C**) Mean temperature, (**D**) maximum temperature, (**E**) relative humidity and (**F**) Normalized different vegetation index (NDVI) at the monthly time scale from January 2000 to December 2018.

Figure 1 displays the annual cycle of malaria incidence rates (MIR) per 100 000 population, precipitation, mean temperature, maximum temperature, relative humidity, and normalized difference vegetation index (NDVI) in Mozambique between 2000 and 2018. There were 88,948,273 malaria cases reported over 18-years period. Between 2001 and 2008, the malaria incidence rate rose from 1081 to 2305 per 100,000 population. This was followed by a decrease from 1874 to 1684 per 100,000 population between 2008 and 2014. From 2015 to 2018, the rate then increased again, reaching 2164 per 100,000 population. The transmission of malaria peaks in summer (Nov-Apr) following the wet season with January having highest malaria cases (Figs. 1A and S2). The wet season lasts from November to April, though most precipitation is concentrated within December to February (Figs. 1B and S2). High precipitation areas include the northern provinces of Cabo Delgado with 89 mm, Niassa with 102 mm, and Nampula with 93 mm, along with the central provinces of Zambezia with 95 mm and Manica with 81 mm, compared to the national average of 77 mm (Figure S7). Mozambique has a consistent seasonal temperature profile with a minimum in May–August (winter) 20.80(± 1.02) and a peak in November–April (summer) 25.94(± 1.09) (Fig. 1C). Malaria seasonality varies across the country, with the peak transmission season occurring earlier in the year in Gaza, Maputo City and Maputo and later in the year in Niassa, Cabo Delgado, Nampula province (Figure S4).

Table 1 displays lag variable selection based on DIC and cross-validated (CV) mean logarithmic score. The selected climate variables were normalized different vegetation index with combined lag 3 and 4; relative humidity with combined lag 5 and 6; precipitation with combined lag 1 and 2; and mean temperature with combined lag 1 and 2. And the third best classified lagged climate variable are normalized different vegetation index at lag 3, relative humidity at lag 6, precipitation at lag 2 and mean temperature at lag 1.

**Table 1:** The top for each lagged climate variables that contributed to the final model, according to deviation information criteria (DIC) and cross-validated (CV) mean logarithmic score.

Variable	Lag	DIC	Coeff	Low	High	CV log score
NDVI	3_4	492,264.139	− 0.540	− 0.778	− 0.302	7.1456
	2	492,271.188	− 0.296	− 0.484	− 0.1085	7.1459
	3	492,272.057	− 0.338	− 0.525	− 0.1501	7.146
RH	5_6	492,168.192	− 0.014	− 0.016	− 0.0113	7.144
	5	492,200.117	− 0.010	− 0.012	− 0.0079	7.145
	6	492,221.486	− 0.0103	− 0.0124	− 0.0083	7.1449
Precipitation	1_2	492,161.093	0.00072	0.0006	0.00085	7.144
	1	492,200.666	0.00082	0.00064	0.00099	7.145
	2	492,212.969	0.00068	0.000508	0.00084	7.14482
TMean	1_2	492,164.423	− 0.057	− 0.0712	− 0.042	7.14452
	2	492,178.306	− 0.036	− 0.047	− 0.0241	7.14454
	1	492,187.326	− 0.034	− 0.045	− 0.0222	7.14504

**Fig. 2:**
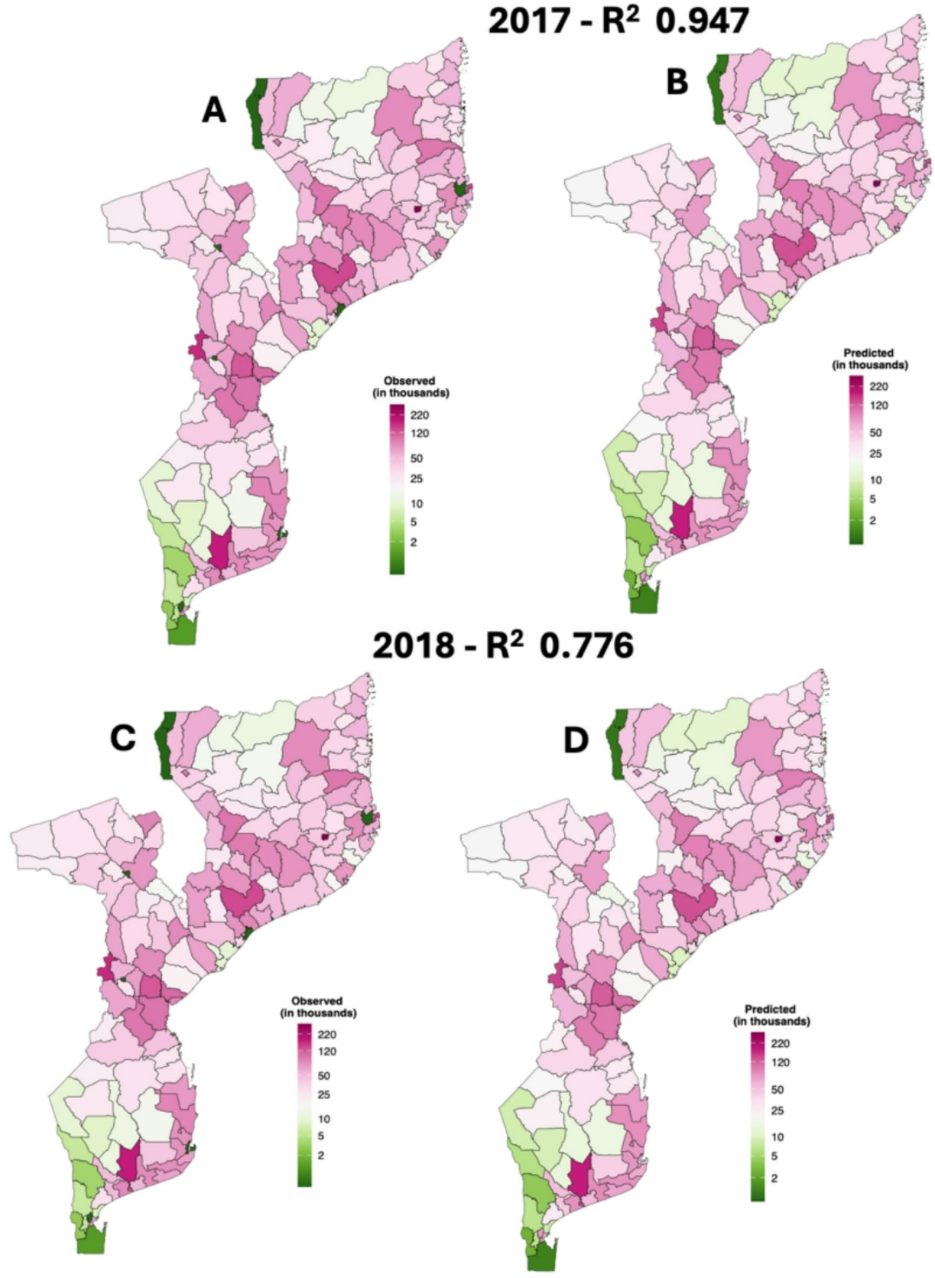
Spatial distribution of observed and predicted malaria cases for 2017 and 2018 in Mozambique. We used ggplot2 in R to create a spatial map visualizing the observed and predicted values for 2017 and 2018. The data is mapped using geom_sf() function, where regions are filled with colors based on the observed and predicted values, scaled using a log-transformed PiYG gradient for better contrast. A black border outlines the regions, while unnecessary plot elements are removed for a clean and focused visualization. The legend, positioned inside the map, enhances clarity by displaying the observed values (in thousands). This map was created using R software (version 4.2.0), which is freely available and can be downloaded from the following link: https://sourceforge.net/projects/rportable/files/R-Portable/4.2.0/.

Figure 2 shows spatial variation in observed and predicted malaria cases between 159 districts in Mozambique. Between 2001 and 2018 a total of 105,397,762 malaria cases were predicted in Mozambique. The predicted malaria cases were 7,644,308 and 7,722,684 in the years 2017, and 2018, while the observed cases were 7,375,745 and 7,369,337 respectively (Fig. 2). The model displayed better predictive accuracy with R^2^ of 94.7% and 77.6% in the years 2017 and 2018 respectively. Observed malaria cases was generally highest in northern and central part of Mozambique, except for some districts in Niassa and Tete province which demonstrated substantially lower malaria cases (Fig. 2A,C). While the predicted malaria cases follow the spatial distribution with observed in the study area (Fig. 2B,D). Estimates of malaria cases were lower than observed cases in southwest Mozambique, particularly certain districts of Gaza, Tete and Niassa provinces (Fig. S13).

**Fig. 3:**
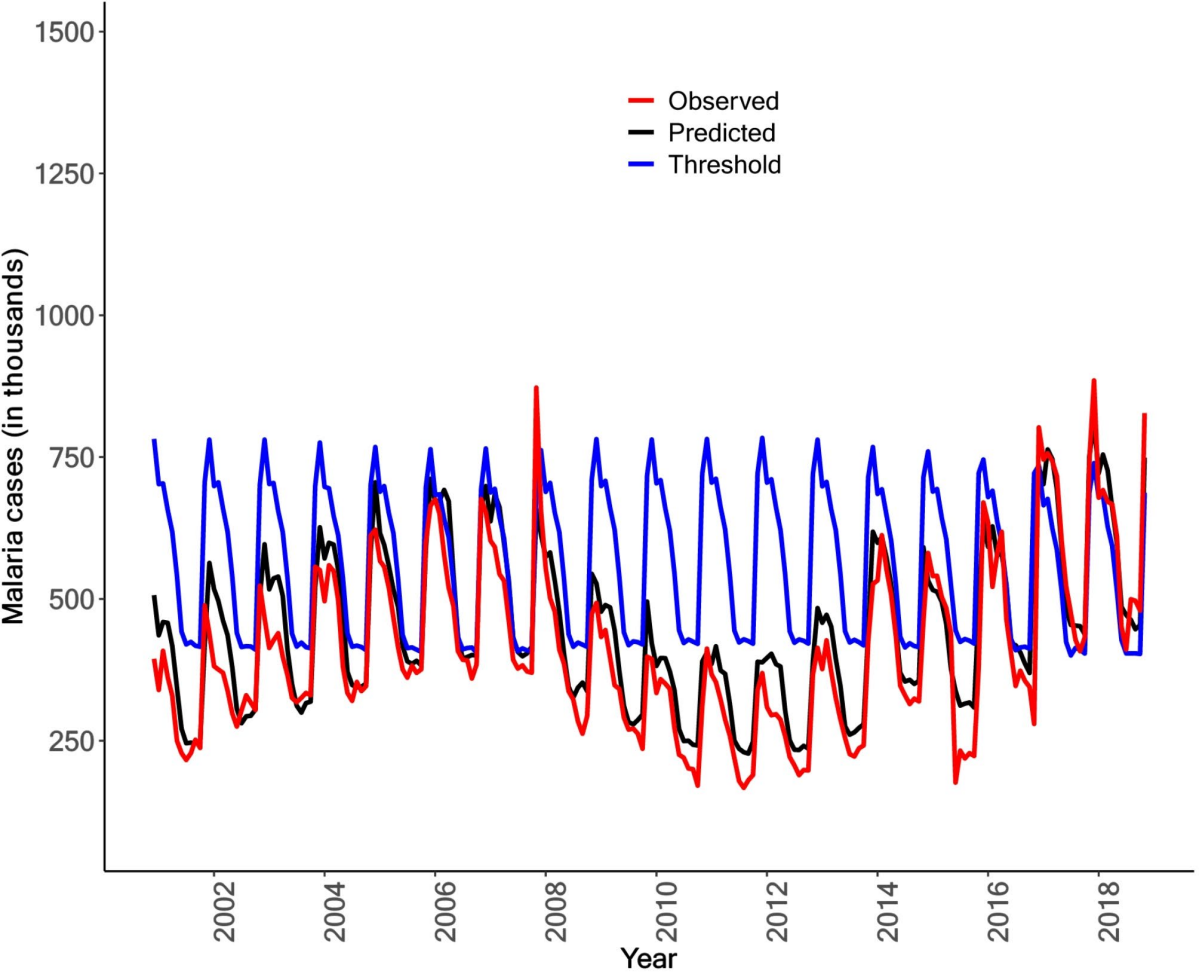
Predicted and observed malaria cases in Mozambique for the period of 2001–2018. The shaded area shows the 95% credible intervals of the predicted cases.

Figure 3 displays the predicted and observed malaria cases in Mozambique for the period of 2001–2018. 95% predicted credible intervals (shaded area) for malaria cases. The model robustly captures the overall trends and spatial temporal heterogeneities in malaria transmissions for Mozambique (Fig. 3). The observed trends are within the model predicted credible intervals (Fig. 3). The contrasting and declining trends for Maputo city, and Maputo were also equally captured (Figure S3). The yearly accuracy statistics for malaria predictions in Mozambique revealed notable variability across the years analyzed (Table S2). Specifically, the coefficient of determination (R-squared) for the predictions was relatively low for 2015 and 2016, recorded at 0.353 and 0.343, respectively. This suggests that the model struggled to capture the variability in malaria incidence accurately during those years. In contrast, the model demonstrated substantially higher accuracy for 2014 and 2017, with R-squared values of 0.959 and 0.947, respectively. These results indicate that the model performed exceptionally well in these years, accounting for nearly all the observed variance in malaria incidence. The discrepancies in prediction accuracy is attributed to factors such as data quality issues, including significant missing data in 2015 and 2016, which were not accounted for using imputation techniques in this analysis (Table S2).

**Fig. 4:**
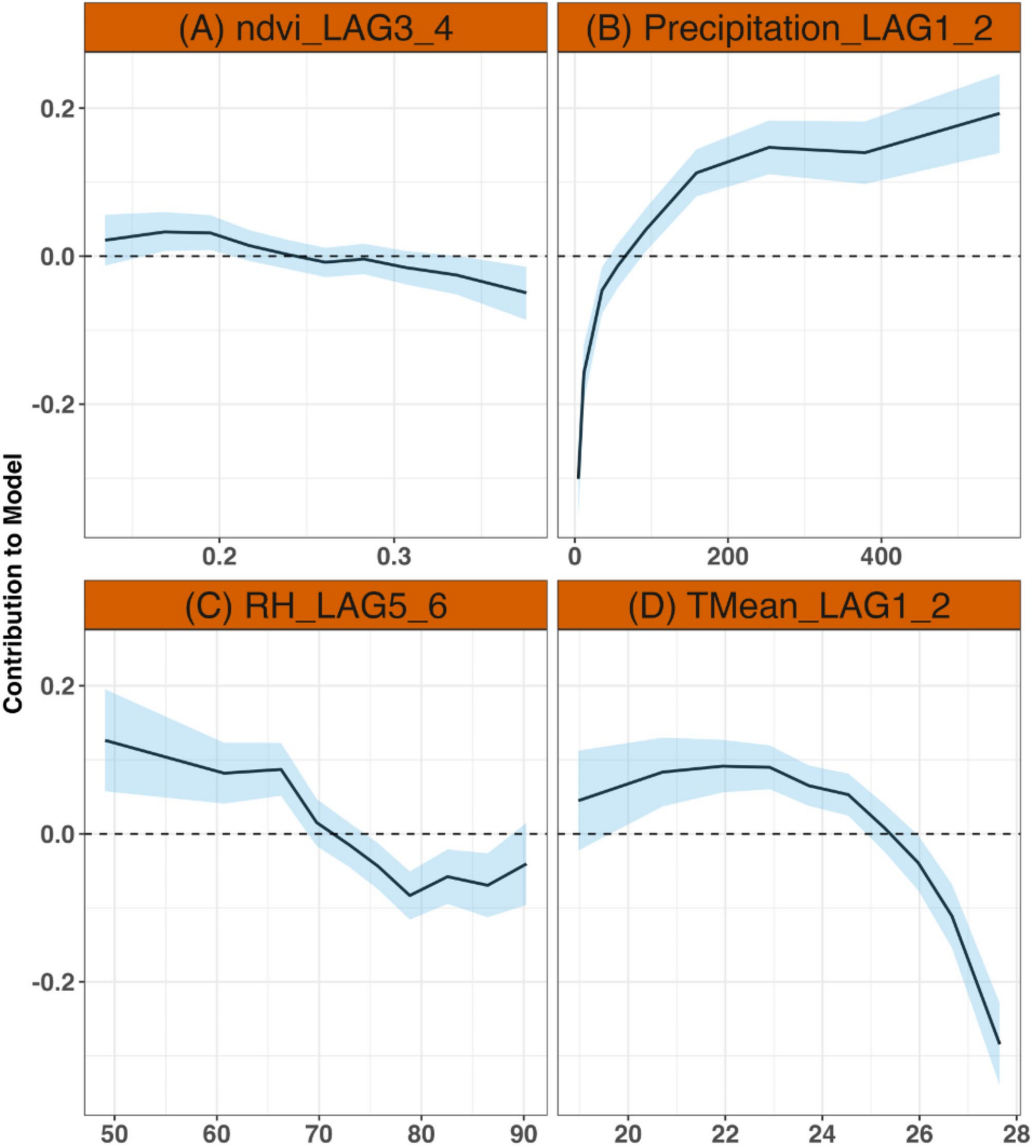
Contribution to the model for each selected climate factors (**A**) normalized different vegetation index at lag3_4, (**B**) precipitation at lag1_2, (**C**) relative humidity at lag5_6 and (**D**) mean temperature at lag1_2. The shaded areas (light blue) correspond to the 95% confidence intervals.

Figure 4 shows the Contribution to the final model for each selected climate factors. We observed high contribution for normalized different vegetation index (NDVI) values between 0.135 and 0.238 (Fig. 4A). We also observed a decreasing contribution to the model for NDVI above 0.238 (Fig. 4A). In Fig. 4B, the contribution was significantly higher at precipitation value above 92.68 mm. The lower contribution occurred at precipitation values below 92.68 mm. Figure 4C displays the contribution to the model for relative humidity (RH). For RH greater than 69.75%, we observed a decrease in contribution to the model while for RH below 69.75% we observed positive contribution to the model. In Fig. 4D, we observed high contribution for mean temperature values below 25.28. The contribution to the model decreased as mean temperature increased (Fig. 4D).

Table 2 shows the forecasting system demonstrated a strong ability to predict malaria cases in Mozambique, with an Area Under the Curve (AUC) of 0.897 (95% CI 0.893–0.901), indicating high overall accuracy. The system’s sensitivity was 0.835 (95% CI 0.827–0.843), meaning it was highly effective at correctly identifying actual malaria cases. Additionally, it achieved a specificity of 0.793 (95% CI 0.787–0.798), reflecting a solid capability to correctly identify non-cases. These metrics highlight the system’s reliability in forecasting malaria outbreaks in the region. The forecasting system perform better in Maputo City (AUC 0.985) than in the whole country (AUC 0.897). In Tete province, the forecasting system demonstrated lower performance, with an AUC of 0.574, compared to the overall performance across the country. This discrepancy is largely attributed to missing data in the region, which likely impacted the model’s ability to accurately capture trends and predict outcomes. The lack of comprehensive and reliable data in Tete may have limited the system’s capacity to generate robust predictions, underscoring the importance of addressing data gaps for improved forecasting accuracy in such areas. The outbreak probability cut-off for outbreak detection varied between 0.051 and 0.338.

**Table 2:** Receiver operating characteristic (ROC) analysis output by province: AUC, cut-off, sensitivity, and specificity with 95% Credible Interval.

Province	Cutoff	AUC (95% CI)	SEN (95% CI)	SPE (95% CI)
Cabo Delgado	0.164	0.796 (0.733–0.859)	0.816 (0.708–0.925)	0.641 (0.568–0.713)
Niassa	0.216	0.827 (0.771–0.883)	0.821 (0.721–0.922)	0.700 (0.629–0.771)
Nampula	0.206	0.792 (0.732–0.851)	0.822 (0.734–0.910)	0.643 (0.565–0.722)
Zambezia	0.208	0.842 (0.779–0.905)	0.806 (0.708–0.905)	0.779 (0.714–0.845)
Tete	0.242	0.574 (0.488–0.660)	0.672 (0.552–0.793)	0.582 (0.505–0.659)
Manica	0.194	0.710 (0.635–0.785)	0.817 (0.719–0.915)	0.603 (0.526–0.679)
Sofala	0.194	0.765 (0.700–0.829)	0.913 (0.832–0.994)	0.559 (0.484–0.633)
Inhambane	0.204	0.656 (0.566–0.746)	0.696 (0.563–0.829)	0.682 (0.612–0.752)
Gaza	0.220	0.717 (0.649–0.785)	0.960 (0.906–1.000)	0.470 (0.394–0.546)
Maputo	0.051	0.687 (0.619–0.754)	0.983 (0.950–1.000)	0.510 (0.431–0.588)
Maputo City	0.338	0.985 (0.973–0.998)	1.000 (1.000–1.000)	0.929 (0.889–0.970)
Mozambique	0.304	0.897 (0.893–0.901)	0.835 (0.827–0.843)	0.793 (0.787–0.798)

*AUC* area under the ROC curve, *SEN* sensitivity, *SPE* specificity.

Table 3 shows model adequacy statistics for different models by increasing the complexity. A baseline model (model 1) was fitted including a monthly and spatial random effect, which accounted for variation in malaria cases. Model 2, selected climate variables, TMean_LAG1_2 + Precipitation_LAG1_2 + RH_LAG5_6 + ndvi_LAG3_4 based on DIC were then added to the baseline model as linear effect. In the final model (model 3), after incorporating monthly and spatial random effects, selected climate variables were added using nonlinear modeling with INLA, employing a random walk of order 1.

**Table 3:** Model adequacy results for models of increasing complexity. The CV mean logarithmic score, and the Deviation Information Criteria (DIC).

Model	Log($\rho_{t} )$	CV log score	DIC
1	Baseline model (season random effect and spatial)	7.1457755	492,283.86
2	Season random effect and spatial and linear effect of the covariates in the model	7.141517	491,965.40
3	Season random effect and spatial and nonlinear effect of the covariates in the model using random walk terms	7.141823	491,961.92

## Discussion

In this study, we developed and evaluated a spatial–temporal prediction model for monthly malaria incidence in Mozambique. The analysis of malaria incidence in Mozambique highlights significant seasonal and spatial variations influenced by climatic factors such as precipitation, mean temperature, maximum temperature, RH, and NDVI. This result aligns with findings from Benin^29^ and Burkina Faso^30^, where malaria incidence is positively associated with mean RH, rainfall, as well as mean and maximum temperatures .

The malaria prediction model demonstrated strong performance, especially for short-term forecasts of 1–2 months ahead. NDVI and RH were found to positively influence malaria incidence at longer lead times of 4–5 months, whereas mean temperature and excess precipitation increased the risk at shorter lead times of 1–2 months. Studies have indicated that precipitation, NDVI, RH and mean temperature are frequently considered significant predictors of malaria incidence^32,33^. A study conducted in Togo^33^ revealed that selected climate factors and their time lags varied across districts and target groups, consistent with other evidence of spatial and temporal variations^34^. In this study, we have outlined the selection of lag variables using DIC deviation information criteria and cross-validated mean logarithmic score. This analysis highlights key climate variables that influence malaria incidence, namely NDVI with lags 3 and 4, RH with lags 5 and 6, precipitation with lags 1 and 2, and mean temperature with lags 1 and 2. The contribution analysis of selected climate variables to the final predictive model indicates that NDVI values between 0.135 and 0.238 had substantial impacts, with diminishing effects observed for values above 0.238. Precipitation values exceeding 92.68 mm significantly enhanced model performance, whereas contributions were lower below this threshold. RH demonstrated a positive contribution to the model below 69.75%, with decreased influence at higher RH levels. Mean temperature values below 25.28 °C had a pronounced impact on the model, which diminished as temperatures increased.

The model demonstrated high predictive accuracy with R^2^ values of 94.7% and 77.6% for the years 2017 and 2018, respectively. Predicted malaria cases closely followed spatial distribution patterns observed in the study area though estimates were notably lower than observed in certain districts of southwest Mozambique. Overall, predicted malaria cases were highest in the northern and central regions of Mozambique and lower in the southern regions, aligning with findings from Colborn’s study^35^. The model effectively classifies high and low malaria seasons, with observed trends generally falling within the model’s predicted credible intervals, demonstrating its reliability. Notably, the model accurately captured both contrasting and declining trends.

Our study shows that the forecasting system demonstrated varying levels of accuracy across Mozambique, performing significantly better in Maputo City compared to the national average, while showing weaker results in Tete province. These regional differences highlight the variability in the model’s effectiveness based on geographic location. The outbreak probability cut-off for detection ranged from 0.051 to 0.338, emphasizing the need for tailored approaches in outbreak surveillance and response strategies. A study in India found that the malaria forecast model effectively predicted *Plasmodium falciparum* incidence in regions with high seasonal and annual variation, particularly in northeastern and northwestern states, such as Orissa, West Bengal, and Rajasthan^36^.

Seasonal prediction models, like the one presented in this study, can help reduce the risk of malaria transmission by providing more preparation time and optimizing the allocation of scarce resources. At present, there are few localized studies in Mozambique that quantify the associations between seasonal climate predictions and malaria risk outcomes. Without a strong evidence base, utilizing climate information for predicting increased malaria risk remains an underutilized opportunity to enhance climate-driven malaria early warning systems.

Interventions implemented in the study area, including insecticide-treated nets (ITNs), prophylactic antimalarial drugs, and indoor residual spraying (IRS), play a critical role in reducing malaria infections in Mozambique. These measures are central to the country’s efforts to combat the disease, as outlined in Mozambique’s 2017–2021 National Malaria Strategic Plan. This strategic plan aims to ensure that at least 85% of the population receives adequate protection against malaria. Key initiatives under this plan include the provision of malaria testing for all suspected cases and treatment for all confirmed cases in accordance with national malaria treatment guidelines^37^. Additionally, the strategy emphasizes targeted interventions to achieve malaria elimination in areas with low and very low transmission. These goals reflect Mozambique’s broader commitment to reducing the malaria burden through comprehensive and equitable health interventions, aligning with both national priorities and global efforts to combat malaria^38^.

The development and implementation of a malaria Early Warning System in Mozambique could represent a groundbreaking strategy in reducing the burden of the disease and lowering infection rates across the country. This innovative system would serve as a proactive tool, enabling health authorities to anticipate outbreaks rather than merely reacting to them. By integrating diverse data streams, such as detailed epidemiological records and real-time climate information, the Early Warning System would provide a comprehensive framework for understanding and predicting malaria transmission patterns. The system’s predictive capabilities would empower public health officials to identify areas and periods of heightened risk, facilitating the timely deployment of targeted interventions. For instance, in regions forecasted to experience outbreaks, resources such as insecticide-treated nets (ITNs), indoor residual spraying (IRS), and community health campaigns could be strategically prioritized. Additionally, the Early Warning System would enhance the logistical planning of diagnostic and treatment supplies, ensuring they reach the most vulnerable populations ahead of anticipated surges in cases. Beyond its immediate benefits in outbreak prediction and response, the Early Warning System could contribute to broader goals of malaria control and elimination by fostering data-driven decision-making and optimizing resource utilization. This transformative approach not only holds promise for significantly reducing malaria incidence in Mozambique but also establishes a scalable and adaptable model that could be replicated in other malaria-endemic countries facing similar challenges.

There are limitations in the study that require attention. Malaria cases in Mozambique are notably high countrywide, with some districts having missing data for the entire period and others experiencing intermittent gaps. The time series is relatively extensive compared to previously analyzed malaria records in Mozambique, yet in 2015, data from July-December are missing. To address this issue in the analysis, we used an imputation method that involved calculating the mean monthly average of the last 5 years of available data to estimate missing values. The model would include data on vector control activities, interventions, and policy changes. Ideally, the model would be enhanced by incorporating comprehensive data on various aspects such as vector control activities, interventions targeting malaria transmission, and changes in health policies. These data would provide a more nuanced understanding of how these factors influence malaria dynamics. By including such information, the model could more accurately simulate and predict malaria incidence, thereby supporting more effective decision-making and resource allocation for malaria control and prevention efforts. In the absence of such data, the model incorporates random effects at monthly and yearly intervals to account for uncertainties arising from these unmeasured aspects of the disease system.

## Conclusion

We constructed a spatial–temporal prediction model that incorporates nonlinear and delayed dynamics inherent in the relationship between malaria and climate variables such as temperature, RH, NDVI and precipitation, in Mozambique. Our findings contribute valuable insights for targeted malaria control and prevention efforts, advocating for adaptive strategies sensitive to regional climate variations and local transmission patterns.

## Methods

### Setting

Figure S1 displays a map of Mozambique, a low-income country located in Southeast Africa, covering an area of 783,000 km^2^, including approximately 4500 km^2^ of maritime area. The main economic activities are agriculture, forestry, and fishing. Mozambican geography is very rich in natural resources though the local population has a low human development index of 0.461^39^ In addition , the country has high levels of social inequality and experiences persistent exposure to extreme weather events such as heavy rainfall, floods, and tropical cyclones^40^.

### Data

District monthly malaria cases from the Mozambique Ministry of Health^41^ for the period 2001–2018 were analyzed in this study. From July to December 2015, there were missing records on malaria cases for the provinces of Cabo Delgado, Gaza, Inhambane, Manica, Maputo, Maputo City, Nampula, Niassa, Sofala, Tete, and Zambezia. Additionally, malaria case data were missing for Inhambane province from May to December 2016, for Tete province in December 2016, and for Zambezia province from November to December 2016. To ensure data reliability and consistency, a quality control process was implemented before imputing missing values for malaria cases. The imputation method involved calculating the mean monthly average of the last 5 years of available data for each respective time point. This approach leverages historical trends to estimate missing values while minimizing bias and preserving seasonality in the dataset. Monthly precipitation, minimum, mean and maximum temperature ($T_{min}$, $T_{mean}$ and $T_{max}$), relative humidity (RH), Normalized Difference Vegetation Index (NDVI) datasets with a spatial resolution of 0.25° × 0.25° for the period of 2001–2018 were sourced from NCEP-reanalysis II^42^. Population datasets from Worldpop, was used as the denominators in the computation of malaria incidence rates and as an offset in the model. WorldPop data is preferred over Mozambique Ministry of Health projections due to its high spatial resolution, frequent updates, consistency across regions, flexibility for detailed spatial analysis, seamless integration with geospatial datasets, and widespread validation in global health research. While Ministry of Health projections are based on local census data, they may be less timely and lack the spatial granularity needed for precise disease modeling.

### Model description

We developed and evaluated a spatio-temporal Bayesian malaria prediction model applying the efficient Integrated Nested Laplace Approximation (INLA) framework.

Model equation

1 $$\log \left( {y_{t} } \right) = \beta_{o} + \varphi_{t^{\prime}\left( t \right)} + \delta_{T^{\prime}\left( t \right)} + f\left( {x_{it} ,l} \right)$$

$y_{t}$ is the number of malaria cases at a month t.

where $\log \left( {y_{t} } \right)$ is the natural logarithm of the dependent variable $y_{t}$ at time t, $\beta_{o}$ is the intercept, monthly, $\varphi_{{t^{\prime } \left( t \right)}}$, and yearly, $\delta_{{T^{\prime } \left( t \right)}}$, random effects (to account for seasonality and interannual variability) and nonlinear exposure lag functions $f\left( {x_{it} ,l} \right)$ of the climate factors $x_{it}$, with lags, l, from 0 to 6 months. The climate factors $x_{it}$ are independent variables or predictors at month $t$ for observation $i$.

Spatial covariance was captured using Besag–York Mollie 2 (bym2) while random walk of order 1 (rw1) was used to account for seasonality and interannual variability. We have considered lag period up to 6 months for each climate factors. We considered the negative binomial distribution due to the overdispersion observed in our data. We developed a baseline model incorporating spatial random effects (BYM2), temporal random effects (RW1), and population as an offset. Variables with superior predictive capacity, as determined by the deviance information criterion (DIC), were included as linear effects in the INLA model. We evaluated different combinations of lag variables through DIC and cross-validation (CV) log scores and included the best-performing combinations as nonlinear effects and random walk terms in the final INLA model, using DIC to assess model fit.

We performed cross-validation, for each combination of month and year. We have assessed the performance of a predictive model by systematically leaving out 1 month from the dataset, training the model on the remaining data, and then using the excluded month to test the model, the process is repeated for each month in the dataset. We computed threshold values for malaria cases in a given district and month based on historical data. The threshold were calculated using 75th percentile from the historical malaria cases.

We calculated exceedance probability of malaria cases over a certain threshold and its implications for outbreak prediction in a specific district in Mozambique. The exceedance probability of the moving outbreak threshold was mapped and cross-checked with actual exceedance data. To optimize the decision trigger for outbreak alerts, a receiver operating characteristic (ROC) curve was generated using binary events that exceeded the threshold. The hit rate (true positive rate or sensitivity) reflects the proportion of correctly predicted outbreaks, while the false alarm rate (1-specificity) represents cases where outbreaks were predicted but did not occur. A receiver operating characteristic (ROC) analysis was performed to evaluate the performance of a model to detect malaria outbreaks based on the computed thresholds. We have computed R-squared statistic, correlation, root mean squared error (RMSE), normalized mean squared error (NMSE), mean absolute error (MAE), and normalized mean absolute error (NMA) to address model predictive accuracy.

The analyses were performed using R software, version 4.2.0^43^, with INLA^44^ packages used for Bayesian modeling and analysis.

## Electronic supplementary material

Below is the link to the electronic supplementary material.

Supplementary Material 1

## Data Availability

The datasets used in this study are accessible from various sources. Malaria datasets are available upon reasonable request by contacting the corresponding author at cjarmando.jose@gmail.com, subject to approval from the Mozambique Ministry of Health’s Disease Surveillance System^39^ (https://www.misau.gov.mz/). The climate data, including precipitation, relative humidity, normalized difference vegetation index, and minimum and maximum temperatures, are freely available from the National Center for Environmental Prediction (NCEP)^40^ at https://psl.noaa.gov/data/gridded/help.html#FTP. Gridded population data are available through WorldPop^41^ at 10.1038/sdata.2017.4.
