# Supplementary material for: Spatio-temporal modelling and prediction of malaria incidence in Mozambique using climatic indicators from 2001 to 2018
Source: Sci Rep. 2025 Apr 8;15:11971. doi: 10.1038/s41598-025-97072-6 (PMC11978812; doi:10.1038/s41598-025-97072-6)
Supplement: Supplementary file 1 — Supplementary Material 1 [file 41598_2025_97072_MOESM1_ESM.pdf]

Supplementary Materials

Spatio-temporal prediction of malaria incidence in Mozambique using climatic indicators: 2001-2018

Chaibo Jose Armando[1*], Joacim Rocklov [2,1], Mohsin Sidat[3], Yesim Tozan[4], Alberto Francisco Mavume[5], Maquines Odhiambo Sewe[1]

***Correspondence:** Chaibo Jose Armando: cjarmando.jose@gmail.com

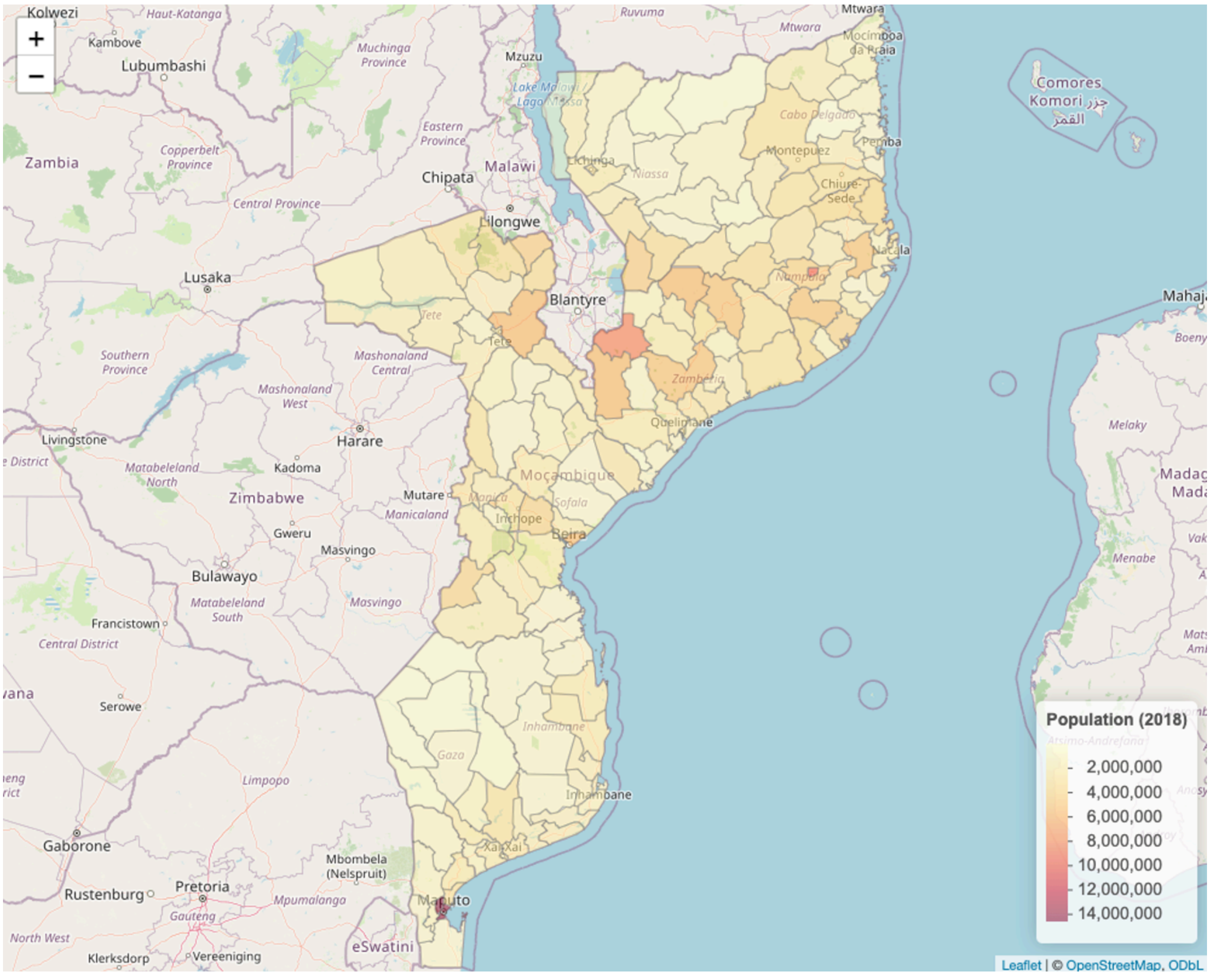

Figure S1 The study area with the distribution of population per district in 2018.

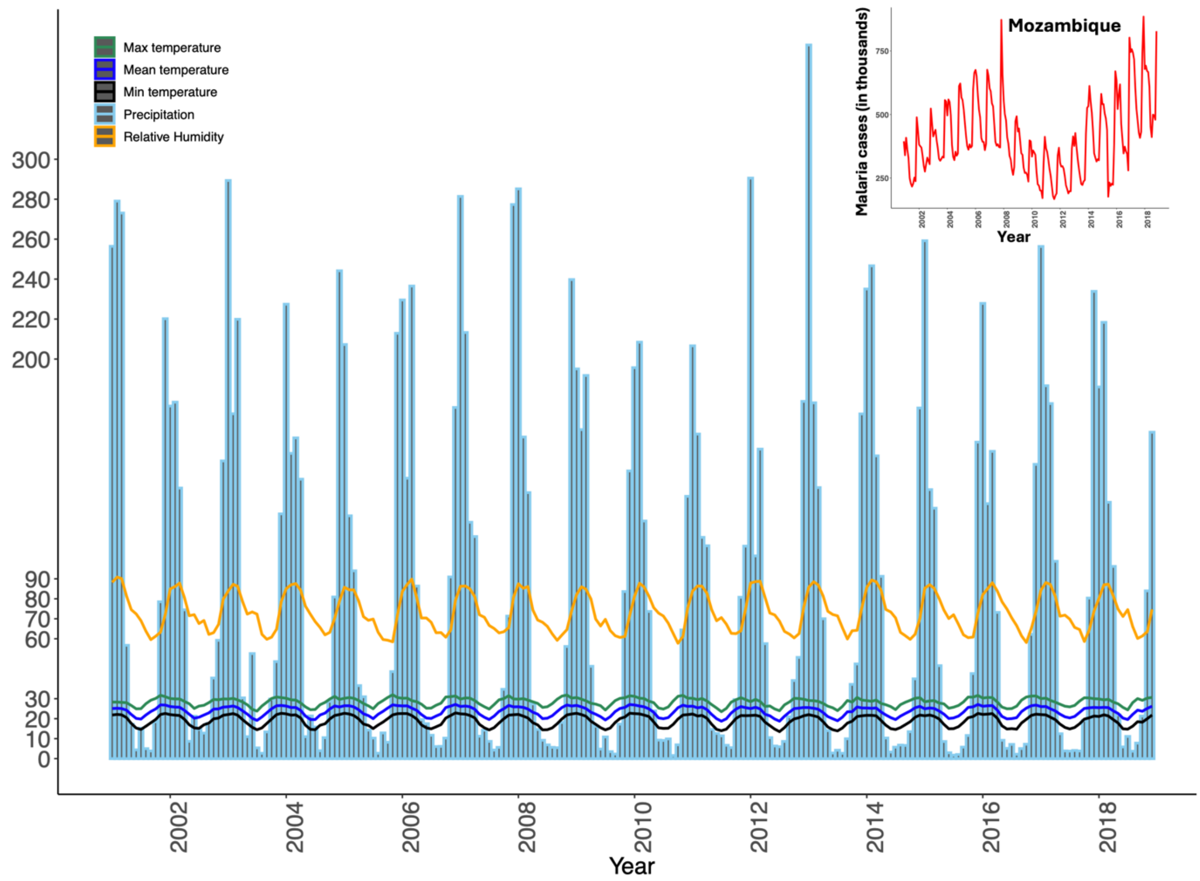

Figure S2 Monthly precipitation, relative humidity, maximum, mean and minimum temperature Climatologically distribution and malaria cases (inset) for Mozambique base period (2001-2018).

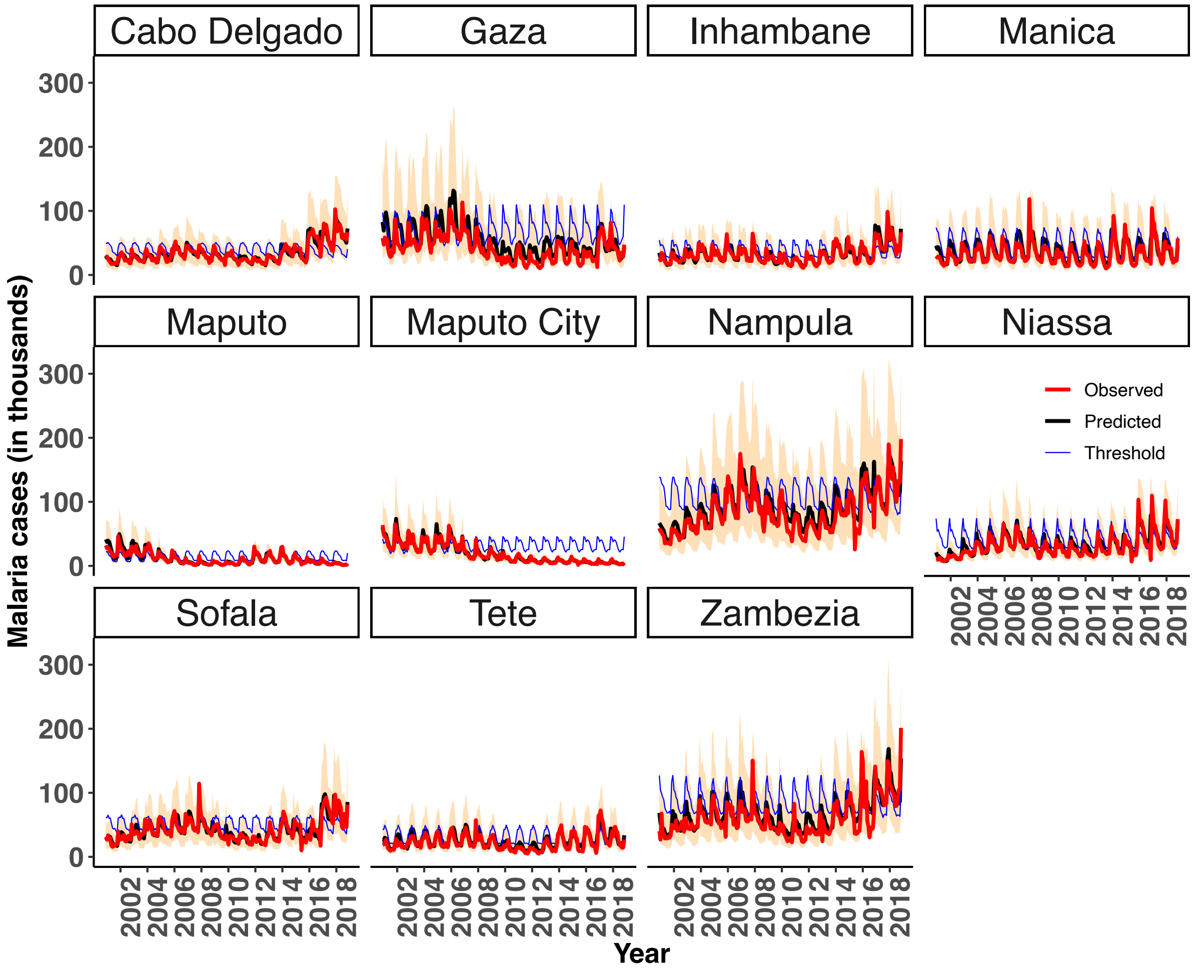

Figure S3 Predicted and observed malaria cases in Mozambican provinces for the period of 2000-2018. 95% predicted intervals (shaded area) for malaria cases.

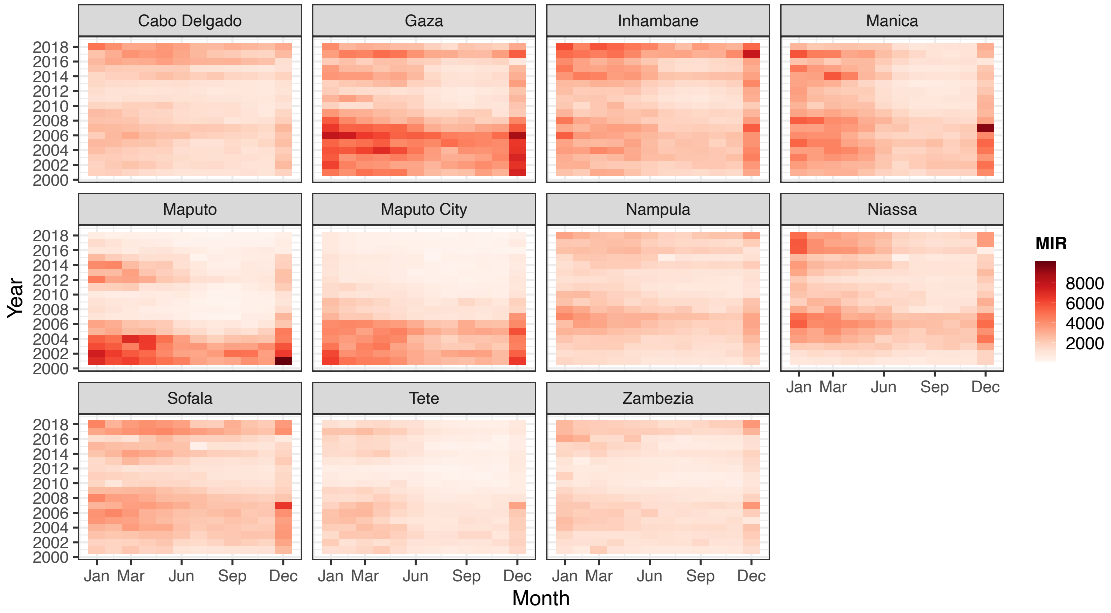

Figure S4 Spatial and temporal variation in malaria incidence rates (per 100 000 population) by province in Mozambique from 2000-2018.

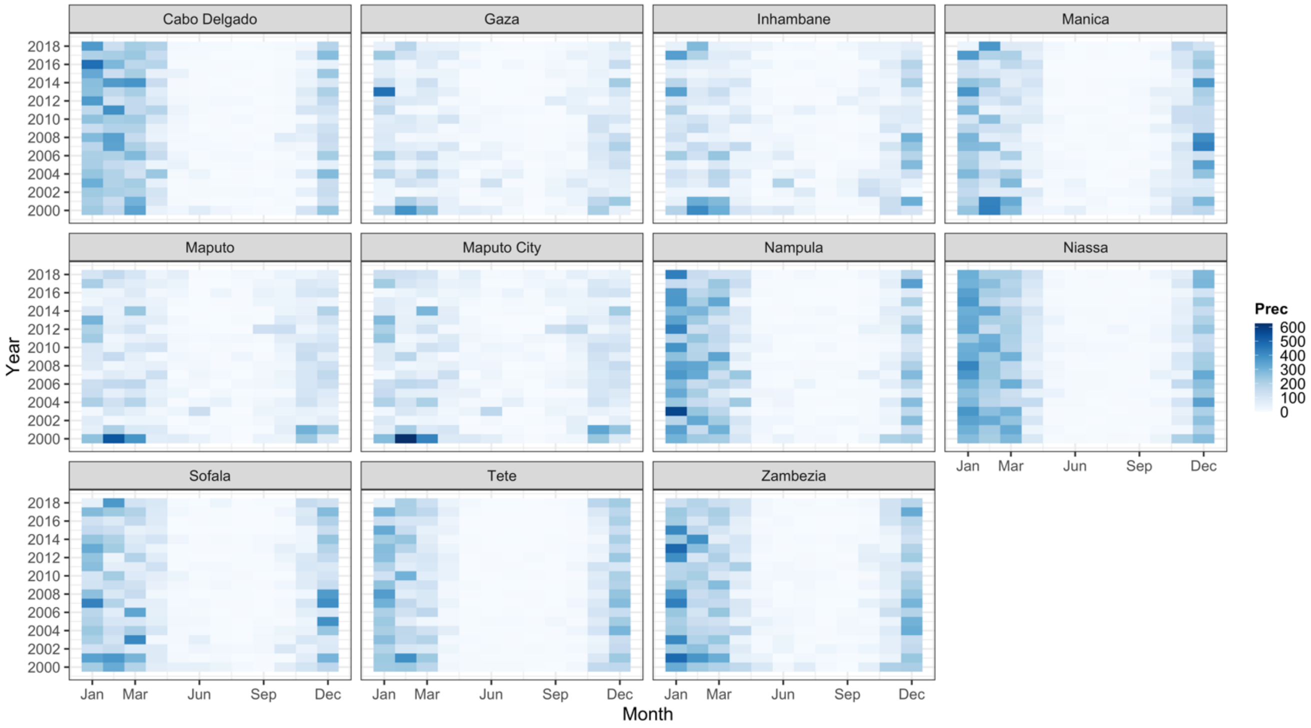

Figure S5 Spatial and temporal variation in precipitation by province in Mozambique from 2000-2018.

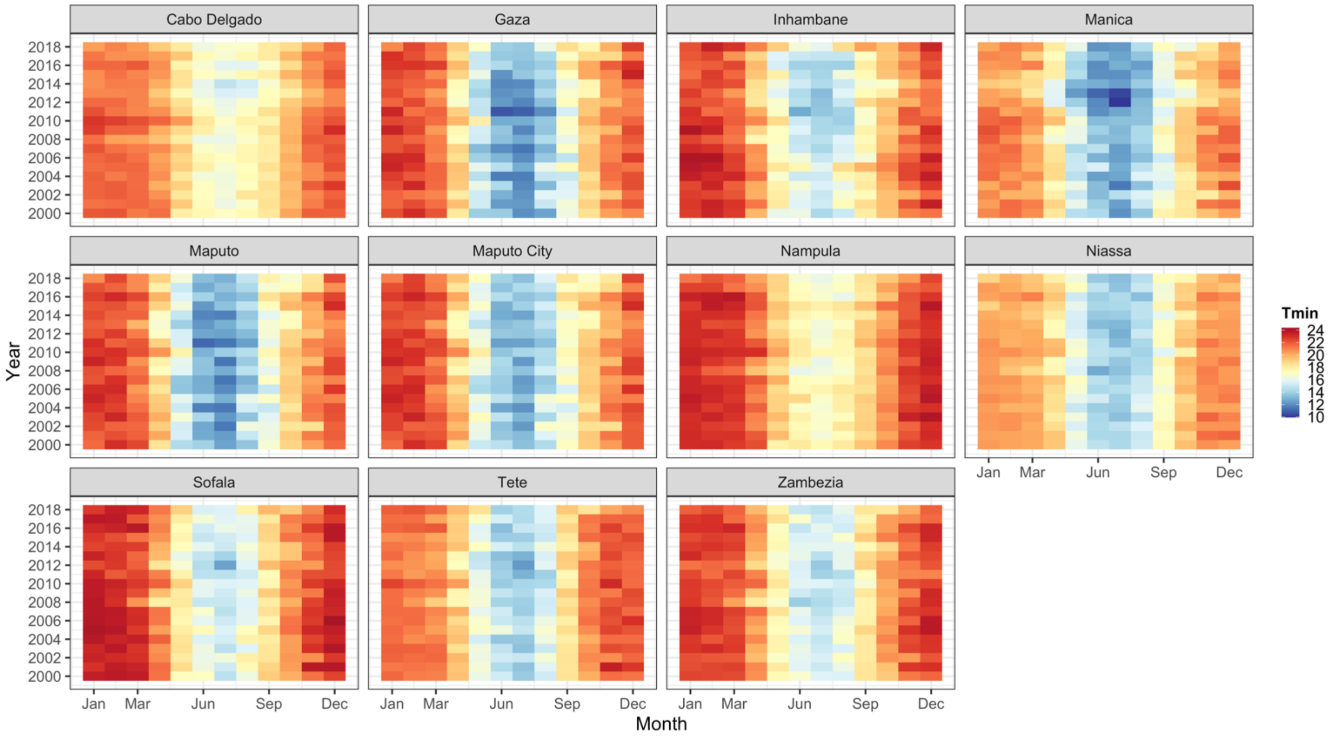

Figure S6 Spatial and temporal variation in minimum temperature by province in Mozambique from 2000-2018.

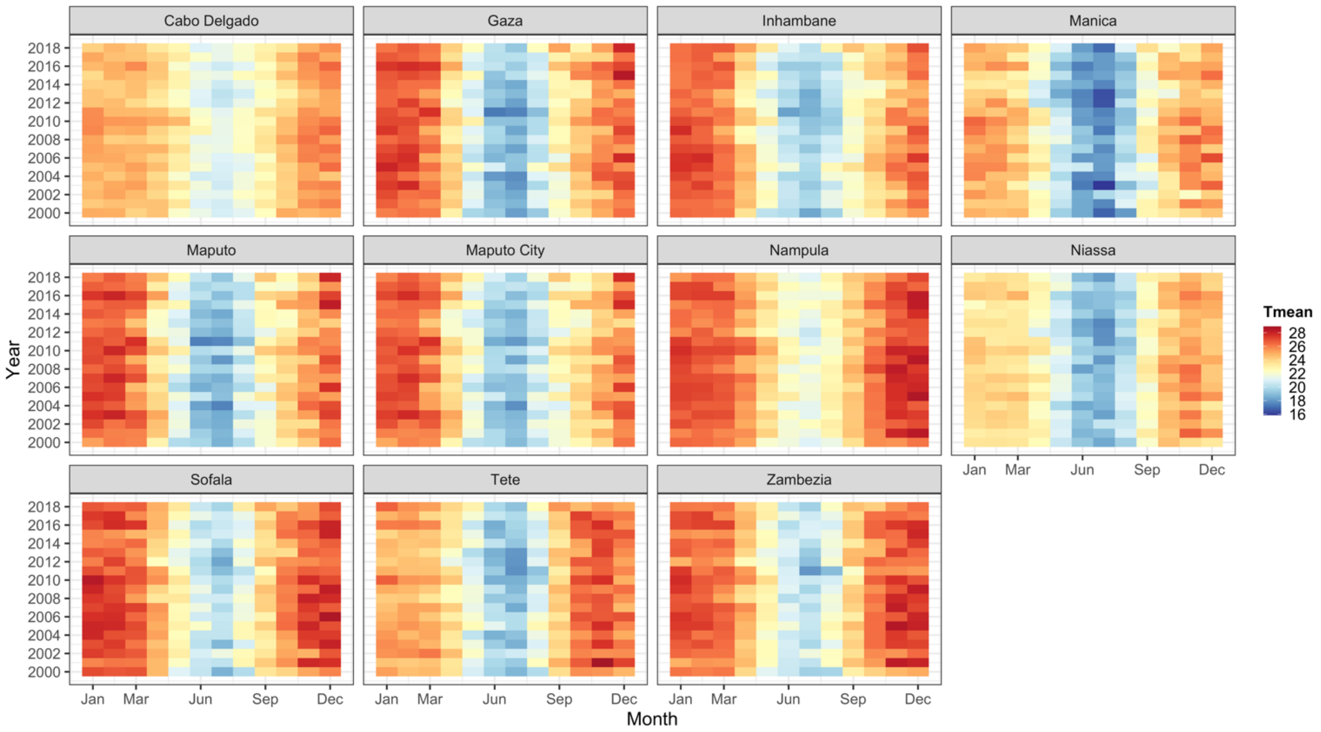

Figure S7 Spatial and temporal variation in mean temperature by province in Mozambique from 2000-2018.

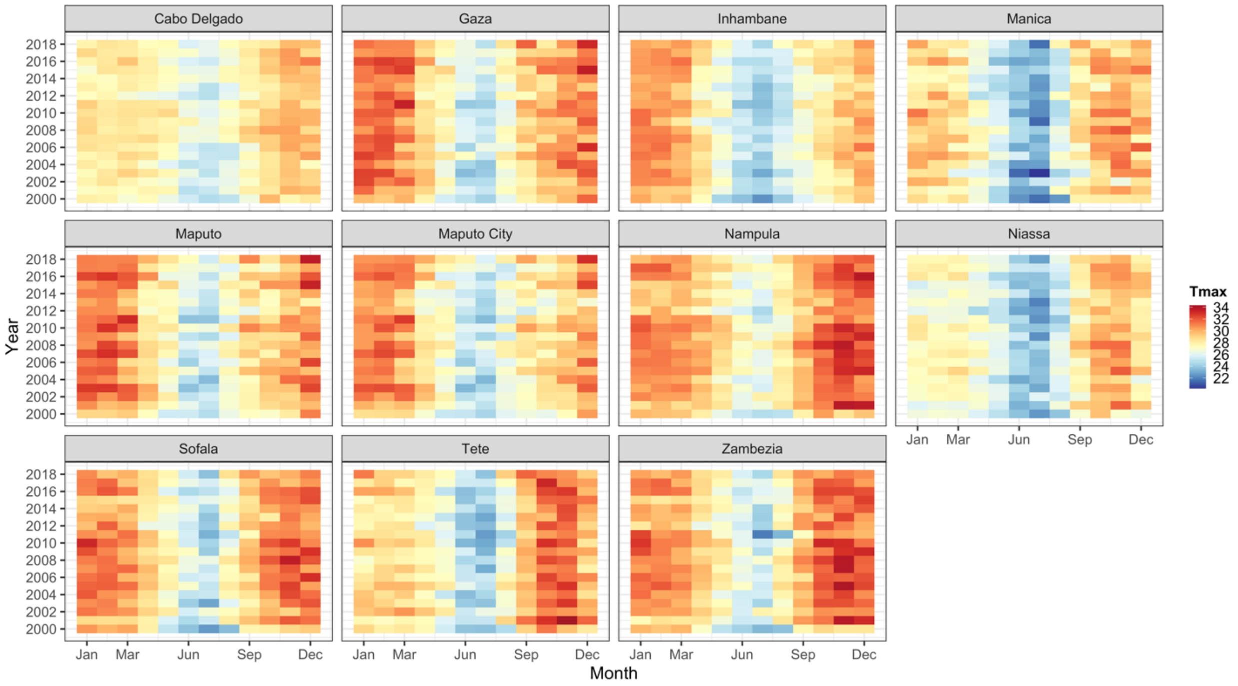

Figure S8 Spatial and temporal variation in maximum temperature by province in Mozambique from 2000-2018.

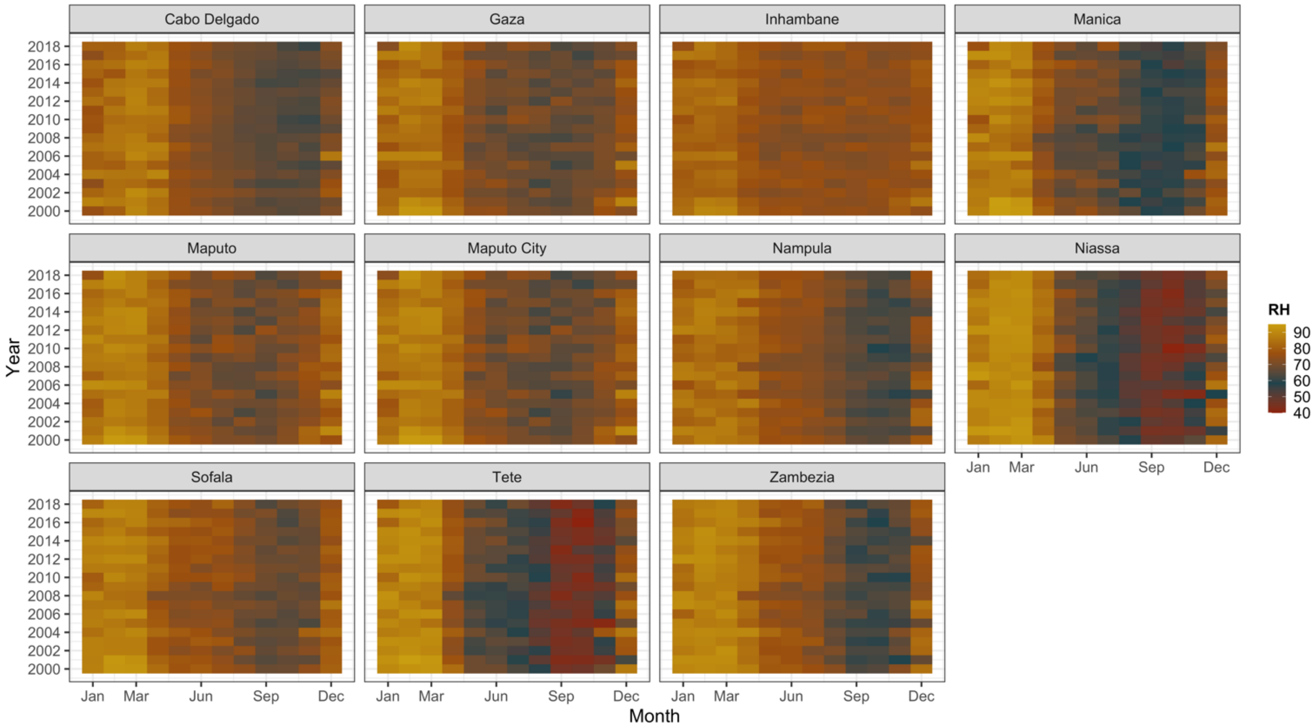

Figure S9 Spatial and temporal variation in relative humidity (RH) by province in Mozambique from 2000-2018.

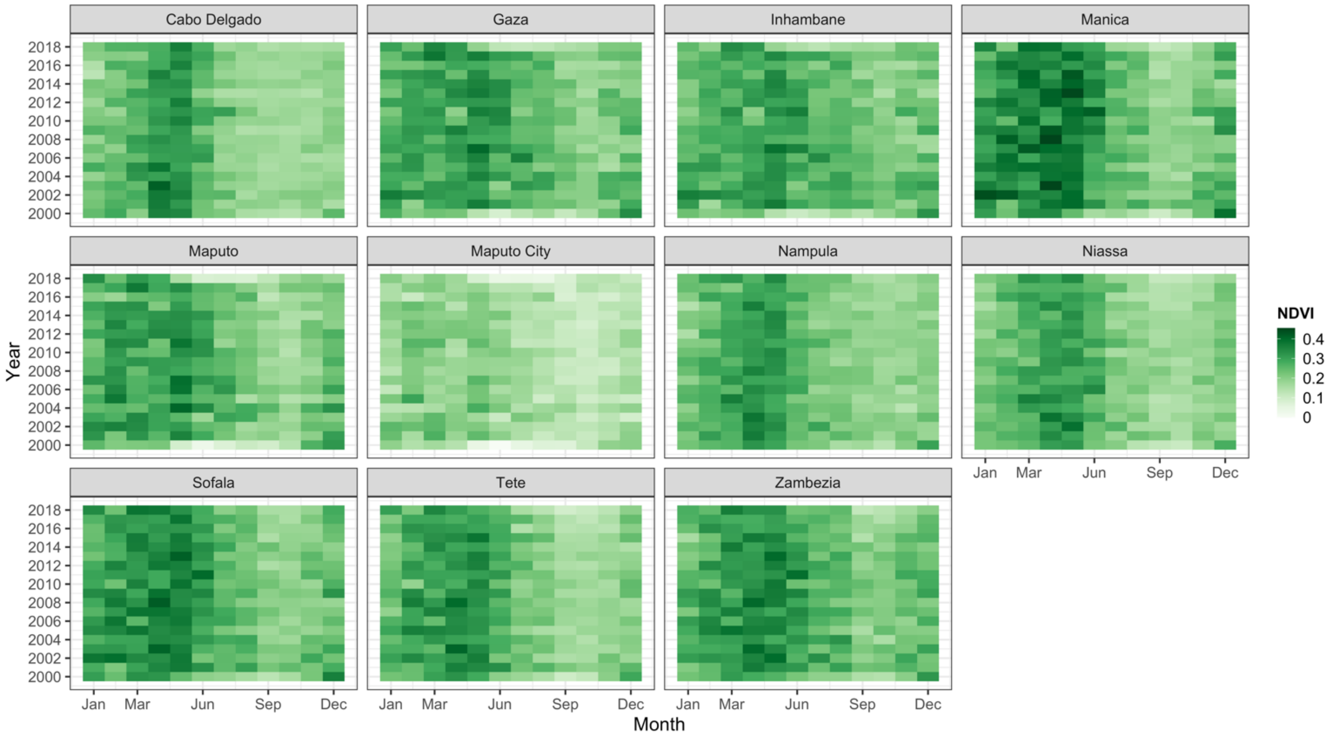

Figure S10 Spatial and temporal variation in normalized different vegetation index (NDVI) by province in Mozambique from 2000-2018.

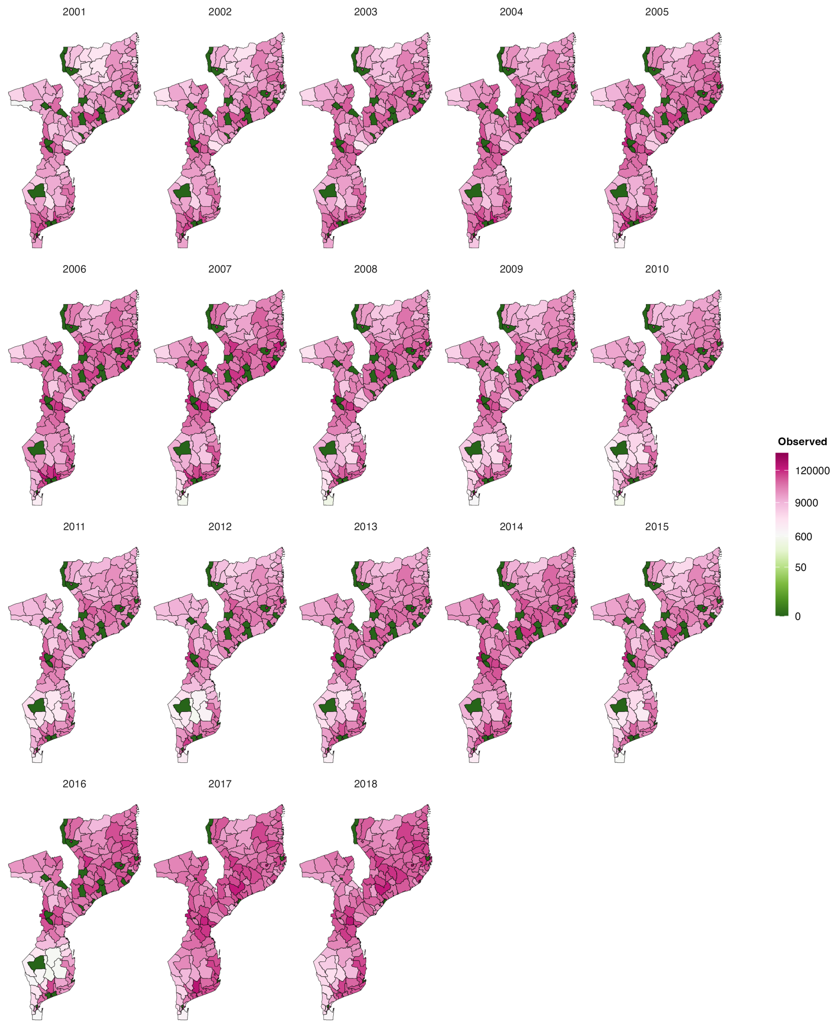

Figure S11 Yearly observed malaria cases for the period of 2001-2018 in Mozambique. We used ggplot2 in R to create a spatial map visualizing the observed values from 2001-2018. The data is mapped using *geom_sf()* function, where regions are filled with colors based on the observed values, scaled using a log-transformed PiYG gradient for better contrast. A black border outlines the regions, while unnecessary plot elements are removed for a clean and focused visualization. The legend, positioned inside the map, enhances clarity by displaying the observed values. This map was created using R software (version 4.2.0), which is freely available and can be downloaded from the following link: https://sourceforge.net/projects/rportable/files/R-Portable/4.2.0/.

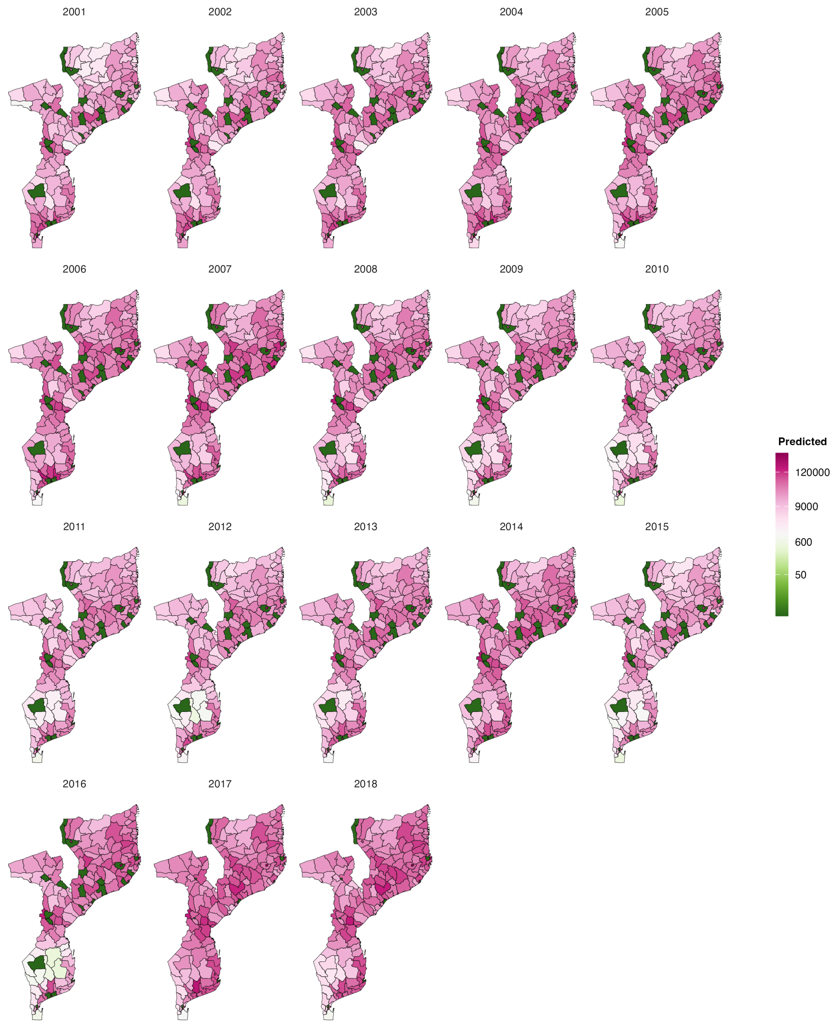

Figure S12 Yearly predicted malaria cases for the period of 2001-2018 in Mozambique. We used ggplot2 in R to create a spatial map visualizing the predicted values from 2001-2018. The data is mapped using *geom_sf()* function, where regions are filled with colors based on the predicted values, scaled using a log-transformed PiYG gradient for better contrast. A black border outlines the regions, while unnecessary plot elements are removed for a clean and focused visualization. The legend, positioned inside the map, enhances clarity by displaying the predicted values. This map was created using R software (version 4.2.0), which is freely available and can be downloaded from the following link: https://sourceforge.net/projects/rportable/files/R-Portable/4.2.0/.

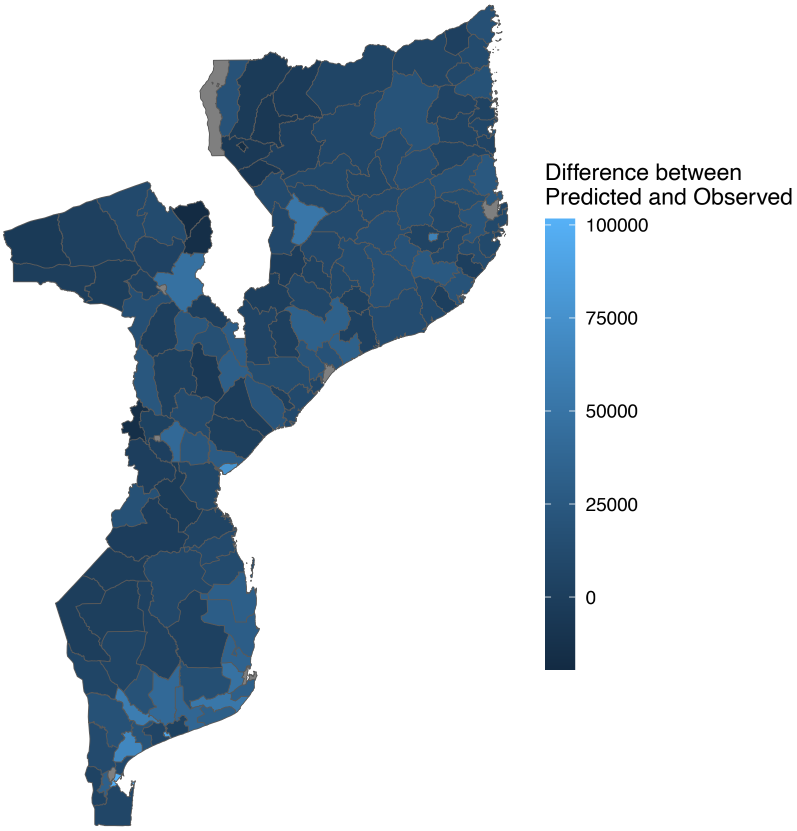

Figure S13 Spatial distribution of difference between predicted and observed malaria cases for the period of 2001-2018 in Mozambique.

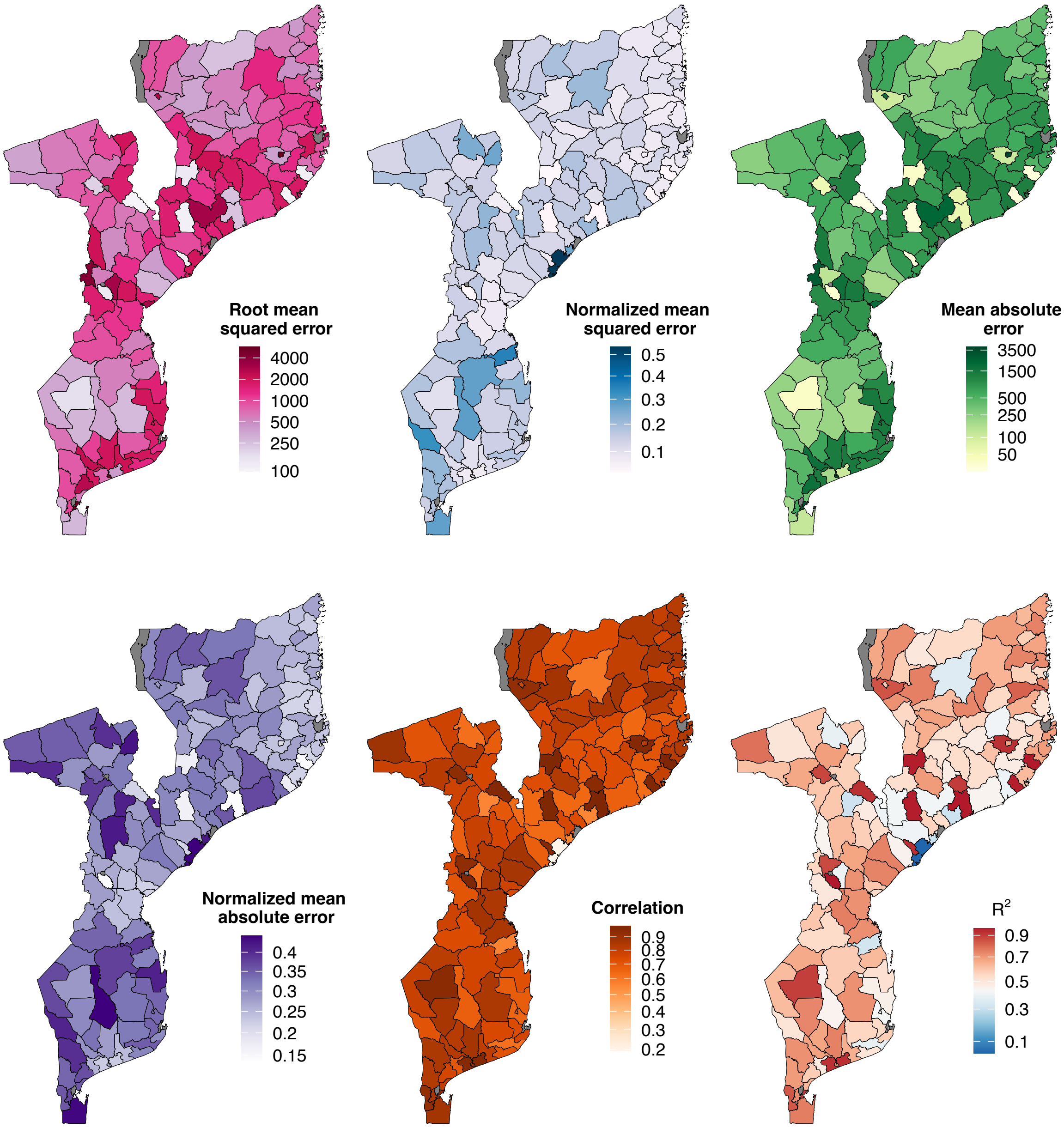

Figure S14 Accuracy statistics for malaria prediction by districts in Mozambique.

Table S1 Accuracy statistics for malaria prediction in Mozambique.

**Accurancy measure**	**V1**
Mean Absolute Error (MAE)	2718.29
Root Mean Squared Error (RMSE)	4340.7
Normalized Root Mean Squared Error (NRMSE %)	28.4
Correlation (r)	0.96

R-squared (R^2)	0.92

Table S2 Yearly accuracy statistics for malaria prediction in Mozambique.

Year	RMSE	Correlation	R^2	MAE	NMSE	NMAE
2001	71237.606	0.743	0.552	57725.028	0.049	0.185
2002	81375.699	0.662	0.439	73549.88	0.051	0.207
2003	83664.48	0.736	0.542	72822.552	0.044	0.186
2004	59048.4	0.887	0.788	50286.746	0.017	0.113
2005	56338.648	0.916	0.838	49688.835	0.014	0.106
2006	68129.783	0.898	0.806	50231.852	0.017	0.097
2007	105171.474	0.73	0.533	70805.031	0.039	0.138
2008	39134.114	0.95	0.903	32021.515	0.008	0.078
2009	37419.937	0.948	0.899	29644.87	0.011	0.085
2010	15251.868	0.982	0.964	11748.052	0.003	0.042
2011	27227.325	0.947	0.897	23799.118	0.009	0.088
2012	41117.214	0.854	0.729	32596.455	0.023	0.122
2013	36914.363	0.929	0.864	30031.333	0.013	0.095
2014	32989.747	0.979	0.959	22336.573	0.005	0.051
2015	240503.522	0.594	0.353	232203.02	0.432	0.889
2016	126390.388	0.585	0.343	78743.454	0.075	0.181
2017	41227.613	0.973	0.947	36154.18	0.004	0.059
2018	82112.37	0.881	0.776	70414.189	0.017	0.115

Table S3 Contribution to the model

Variable	lag	DIC	coeff	low	high	CV log score
ndvi	3_4	492264.139	-0.54005	-0.7777	-0.3023	7.1456
ndvi	2	492271.187	-0.2962	-0.4841	-0.1085	7.1459
ndvi	3	492272.057	-0.3378	-0.5255	-0.1501	7.1457
ndvi	5	492279.126	-0.2040	-0.3926	-0.0154	7.1461
ndvi	1_2	492279.926	-0.0934	-0.3335	0.1466	7.1456
ndvi	6	492281.577	0.0223	-0.1691	0.21390	7.1460
ndvi	1	492282.789	0.1870	-0.0025	0.3765	7.1463
ndvi	4	492296.921	-0.3248	-0.5123	-0.1372	7.1460
Ndvi	5_6	492310.810	-0.1570	-0.3999	0.0858	7.1462
RH	5_6	492168.192	-0.0137	-0.01614	-0.0113	7.1443
RH	5	492200.117	-0.0099	-0.0119	-0.0078	7.1450
RH	6	492221.486	-0.0103	-0.0125	-0.0082	7.1449
RH	4	492227.647	-0.0078	-0.00985	-0.00579	7.1452
RH	3	492235.787	-0.00617	-0.0082	-0.00416	7.1455

RH	3_4	492240.159	-0.00932	-0.01166	-0.00698	7.1452
RH	1	492276.213	0.0047	0.00271	0.0068	7.1460
RH	2	492305.573	-0.00065	-0.00269	0.00137	7.14607
RH	1_2	492309.140	0.00276	0.00039	0.00515	7.14618
Precipitation	1_2	492161.093	0.000729	0.00061	0.00085	7.14401
Precipitation	1	492200.666	0.000819	0.000646	0.000992	7.1448
Precipitation	2	492212.969	0.000678	0.000508	0.000848	7.1448
Precipitation	3	492246.860	0.000360	0.000191	0.000530	7.1455
Precipitation	3_4	492263.217	0.000277	0.000159	0.000395	7.1460
Precipitation_chirps	1	492271.212	0.00033	0.00017	0.00049	7.1461
Precipitation	5_6	492272.919	-0.000202	-0.00032	-0.000836	7.1460
Precipitation_chirps	5	492277.353	-0.00017811893	-0.00033	-0.000227	7.1459
Precipitation	4	492277.640	0.000206	0.000384	0.0003752	7.1460
Precipitation_chirps	3_4	492281.780	-0.000765	-0.00018	3.27384	7.14622
Precipitation_chirps	2	492282.031	0.00018	0.000276	0.000344	7.14592
Precipitation	6	492290.098	-0.00036	-0.00052	-0.000184	7.14571
Precipitation_chirps	1_2	492291.711	0.000250	0.000139	0.000361	7.14571
Precipitation_chirps	4	492303.383	-0.000125	-0.000281	0.000304	7.1458
Precipitation_chirps	6	492304.098	-0.000719	-0.000227	0.000833	7.1458
Precipitation_chirps	5_6	492304.124	-0.00012	-0.000229	-0.000118	7.1459
Precipitation_chirps	3	492304.583	-0.000364	-0.000194	0.000121	7.1460
Precipitation	5	492305.718	-0.00066	-0.000234	0.000101	7.1460
TMean	1_2	492164.422	-0.0566	-0.071255	-0.04203	7.1445
TMean	2	492178.305	-0.03568	-0.047309	-0.02408	7.1445
TMean	1	492187.326	-0.03372	-0.045270	-0.02218	7.1450
tmax	1_2	492221.130	-0.02912	-0.039838	-0.01841	7.1450
tmax	2	492233.282	-0.02154	-0.030272	-0.01284	7.1454
tmean	2	492246.431	-0.01098	-0.02236	0.00040	7.1456
tmax	1	492254.102	-0.01669	-0.02542	-0.00798	7.1456
TMean	3_4	492257.202	-0.00701	-0.02109	0.006919	7.1456
tmean	1_2	492264.655	-0.01475	-0.02896	-0.00067	7.1457
tmin	6	492264.831	0.00326	-0.00748	0.01407	7.1458
tmin	5_6	492267.892	-0.00478	-0.01737	0.00782	7.1458
tmean	6	492268.196	0.01762	0.00611	0.02917	7.1458
TMean	3	492269.321	-0.0088	-0.02041	0.00275	7.1457
tmean	4	492270.106	0.01453	0.003279	0.02567	7.1462
tmin	3	492270.564	0.00557	-0.00535	0.01645	7.1457
tmax	4	492271.002	0.01045	0.001755	0.01911	7.14598
TMean	5	492275.471	-0.01117	-0.02237	0.00019	7.14605

TMean	6	492276.330	0.01163	0.000202	0.02310	7.14613
tmean	1	492276.511	-0.00774	-0.01911	0.00353	7.14616
tmean	5	492277.769	-0.00201	-0.013047	0.009027	7.14561
tmin	1_2	492277.871	0.01878	0.005677	0.031815	7.14608
tmin	3_4	492279.143	0.01167	-0.00110	0.024280	7.14601
TMean	4	492282.770	0.00075	-0.01056	0.012069	7.14626
tmean	5_6	492282.931	0.01034	-0.00324	0.023994	7.14620
tmax	6	492285.822	0.01886	0.00984	0.027914	7.14563
tmax	3	492285.985	0.00033	-0.00827	0.008954	7.14607
tmean	3	492289.488	0.00411	-0.00695	0.015123	7.14596
tmax	3_4	492290.840	0.00857	-0.00208	0.019219	7.14614
tmin	4	492295.672	0.00970	-0.00091	0.020281	7.14614
tmax	5_6	492296.739	0.0175	0.00661	0.028571	7.14590
tmean	3_4	492297.585	0.01355	0.00029	0.026736	7.14638
tmin	2	492299.301	0.01322	0.00226	0.024085	7.146375
tmin	5	492300.986	-0.0103	-0.0209	0.000126	7.145871
TMean	5_6	492305.182	0.00027	-0.0134	0.014071	7.146100
tmax	5	492306.898	0.00424	-0.0044	0.012948	7.145874
tmin	1	492315.982	0.00984	-0.00084	0.020487	7.14628
